# EIF4A3-induced circular RNA PRKAR1B promotes osteosarcoma progression by miR-361-3p-mediated induction of FZD4 expression

**DOI:** 10.1038/s41419-021-04339-7

**Published:** 2021-10-29

**Authors:** Zhen-hua Feng, Lin Zheng, Teng Yao, Si-yue Tao, Xiao-an Wei, Ze-yu Zheng, Bing-jie Zheng, Xu-yang Zhang, Bao Huang, Jun-hui Liu, Yi-lei Chen, Zhi Shan, Pu-tao Yuan, Cheng-gui Wang, Jian Chen, Shu-ying Shen, Feng-dong Zhao

**Affiliations:** 1 Department of Orthopaedic Surgery, Sir Run Run Shaw Hospital, Zhejiang University School of Medicine, Hangzhou, China; 2 Key Laboratory of Musculoskeletal System Degeneration and Regeneration Translational Research of Zhejiang Province, Hangzhou, China; 3 Department of Orthopedics, 2nd Affiliated Hospital, School of Medicine, Zhejiang University, Hangzhou, China

**Keywords:** Bone cancer, miRNAs

## Abstract

Emerging evidence indicates that circRNAs are broadly expressed in osteosarcoma (OS) cells and play a crucial role in OS progression. Recently, cancer-specific circRNA circPRKAR1B has been identified by high-throughput sequencing and is recorded in publicly available databases. Nevertheless, the detailed functions and underlying mechanisms of circPRKAR1B in OS remains poorly understood. By functional experiments, we found that circPRKAR1B enhanced OS cell proliferation, migration, and promotes OS epithelial–mesenchymal transition (EMT). Mechanistic investigations suggested that circPRKAR1B promotes OS progression through sponging miR-361-3p to modulate the expression of FZD4. Subsequently, we identified that EIF4A3 promoted cirPRKAR1B formation through binding to the downstream target of circPRKAR1B on PRKAR1B mRNA. Further rescue study revealed that overexpression of the Wnt signalling could impair the onco-suppressor activities of the silencing of circPRKAR1B. Interestingly, further experiments indicated that circPRKAR1B is involved in the sensitivity of chemoresistance in OS. On the whole, our results demonstrated that circPRKAR1B exerted oncogenic roles in OS and suggested the circPRKAR1B/miR-361-3p/FZD4 axis plays an important role in OS progression and might be a potential therapeutic target.

## Introduction

Osteosarcoma (OS), a neoplasm stemming from mesenchymal stem cells, is one of the most malignant cancers among children and adolescents [1, 2]. In the past 30 years, with the development of neoadjuvant chemotherapy and surgical technology, the overall rate of survival among patients with OS has improved, but patients with pulmonary metastases have poor survival [3]. In order to maximise the therapeutic effectiveness in OS, it is important to develop biomarkers for early diagnosis and novel therapeutic targets. Hence, there is an urgent need for understanding the molecular mechanism of OS progression.

Circular RNAs are noncoding RNAs with no 5′ cap or a 3′ poly (A) tail, and they are not digested by RNA exonucleases, unlike linear RNA [4–6]. Increasing reports indicate the crucial role played by many circRNAs in the development and progression of tumours and other diseases, including osteosarcoma [7, 8]. Researchers have identified many effects of circRNAs, including sponging miRNAs, regulating protein translation, regulating gene transcription and binding to RNA-binding proteins (RBPs) [9, 10]. Most commonly reported is acting as competitive endogenous RNAs (ceRNAs), suppressing miRNA activity and thereby regulating the expression and biological functions of downstream target genes [11]. RNA binding proteins (RBPs), such as QKI and EIF4A3, play an important role in regulating the generation of circRNAs [12, 13]. Recently, cancer-specific circRNA circPRKAR1B has been identified by high-throughput sequencing and is recorded in publicly available database. For examples, circPRKAR1B was reported to act as miR432-5p sponge and promote breast cancer progression by targeting miR432-5p/E2F3 axis [14]. CircPRKAR1B promotes proliferation, migration and invasion of breast cancer cells through upregulating CBX4 via sponging miR-515-5p [15]. CircPRKAR1B was developed and validated to improve the prognostic stratification for patients with colon cancer [16]. Therefore, circPRKAR1B plays an important role in the occurrence and development of cancers. However, it remains imperative to investigate whether circPRKAR1B mediated osteosarcoma progression.

Frizzled class receptor 4 (FZD4), a class Frizzled G protein-coupled receptor (GPCR) also known as a WNT receptor, bears structural similarity to G protein-coupled receptors and activates diverse intracellular signalling pathways [17, 18]. FZD4 is known as an activating factor for Wnt/β-catenin signalling [17]. WNT/Frizzled receptor (FZD) signalling pathways are pivotal for physiological and pathophysiological processes. WNT activates FZD results in the formation of a ternary WNT/FZD/LRP complex that recruits axin and inhibits the destruction complex. Consequently, β-catenin accumulates in the cytoplasm and facilitate their translocation to the nucleus, where it recruits T-cell factor/lymphoid-enhanced factor (TCF/LEF), thus upregulating the downstream genes of WNT [19]. Numerous reports have shown that the Wnt/β-catenin pathway affects cell proliferation and tumorigenesis [20, 21]. Furthermore, the activation of the Wnt/β-catenin pathway can facilitate cell proliferation, migration and invasion, thus promoting OS progression and effectively endowing OS cells with an EMT-like phenotype [22, 23].

In this research, we performed numerous functional and molecular experiments to determine the underlying mechanism of circPRKAR1B in OS progression. Our results highlight a novel molecular mechanism underlying the tumorigenicity and metastasis of OS and suggest that the circPRKAR1B/miR-361-3p/FZD4 axis plays an important role in OS progression and might be a potential therapeutic target.

## Methods

### Ethical approval

All experiments were performed with the approval of the Sir Run Run Shaw Hospital Ethics Committee and followed the Guidelines for Care and Use of Laboratory Animals under the National Institutes of Health.

### Cell culture

The study adopted five OS cell lines (MG63, SAOS, HOS, 143B, U20s), HFOB1.19, and HEK-293T. We maintained cells at 37 °C in Dulbecco’s modified Eagle’s medium (DMEM) containing 10% fetal bovine serum (FBS) with 5% CO_2_.

### Plasmid construction and cell transfection

SiRNAs were provided by RiboBio. MiRNA mimics and inhibitors were purchased from GenePharma (Shanghai, China). Lentivirus miR-361-3p sponge and lentivirus-sh-circPRKAR1B came from HanBio (Shanghai, China). Lipofectamine iMAX (Invitrogen) was used to transfection.

### Subcutaneous xenograft tumour and lung metastasis models

4-week-old male nude mice were randomly applied for the Subcutaneous xenograft tumour as well as lung metastasis models. A total of 5 × 10^6^ 143B stable cells under transfection of sh-circPRKAR1B or the co-transfection of sh-circPRKAR1B and miR-361-3p sponge received subcutaneous injection or tail vein injection (143B had the label of a luminescent dye and GFP). Tumour volume was calculated by the equation *v* = *ab*^2^/2.

### Transwell migration assay

12-well transwell chambers were utilised to evaluate invasion and migration capability of 143B and HOS cells. For migration assays, cells were re-suspended with FBS-DMEM and seeded into upper chambers with a density of 5 × 10^4^ cells/well. A total of 500 ml DMEM was added to the lower chambers that contained 10% FBS. Following 24 h of culture with different treatment, part of cells will migrate from the upper to the lower chamber. Then the chambers were washed, and 4% paraformaldehyde was used to fix the washed chambers for 20 min. OS cell lines received staining treatment by using 1% crystal violet for about 15 min. Afterwards, wash the chambers by PBS for five times and wiped off the cells which were detained in the upper side. Finally, images were captured by microscopy.

### CCK-8 assay

CCK8 assay assisted in detecting the cell viability exhibited by transfected 143B and HOS cells. Briefly, transfected 143B and HOS cells were incubated with 4 × 10^3^ cells in the 96-well plates. After 0, 24, 48, or 72 h, the medium of each well were replaced with 100 μL DMEM including 10% CCK8 reagent (Sigma-Aldrich, St. Louis, MO, USA). Then we employed ELX800 absorbance microplate reader (BioTek Instruments) to measure the absorbance value exhibited by each well at 450 nm.

### Colony-formation assay

Transfected 143B and HOS cells were seeded in 12-well plates at 1 × 10^3^ cells. After about 7 days of culture, 4% paraformaldehyde was used to fix colonies for 20 min, and the fixed colonies received another 20 min of staining at room temperature. The count of colonies was then counted as well as analysed after washing by PBS.

### Soft agar colony formation assay

We inoculated transfected HOS and 143B cells in semisolid agar medium (0.6% agarose/PBS bottom layer with a 0.5% agarose/PBS culture medium in a 12-well plate) at 1 × 10^4^ cells. An inverted microscope (Nikon) helped to photo images on days 0, 3 and 6, respectively.

### EdU assay

Firstly, we seeded 143B and HOS cells in 6-well plates with a suitable density. After adherence, OS cells were starved for about 8 h with FBS-free DMEM to synchronise cell cycle of each wells. Then cells were transfected with specific siRNA or plasmid. After that, the medium of each wells were replaced with fresh DMEM containing 10 μM EdU and cultured at 37 °C, with 5% CO_2_ for another 2 h. Then 4% paraformaldehyde was used to fix the cells for 15 min, and the fixed cells received 20 min of incubation with 0.3% Triton-X-100. Afterwards, cells were cultured with Click reaction buffer for 25 min away from light. Then Hoechst dye 33342 was employed to stain the nuclei. We calculate the cellular proliferation rate in terms of the instructions of the manufacturers (BeyoClick™EdU-488 Cell Proliferation Detection Kit, Beyotime Biotechnology).

### Flow cytometry

We inoculated 143B and HOS cells into 6-well plates with a suitable density. Then the cells were exposed to different transfection for 48 h after they were completely adherent. For cell apoptosis assay, cells were digested, centrifuged, and washed by PBS. Subsequently, we mixed each sample using Annexin V-PE/ 7AAD kit (BD Biosciences, San Diego, CA, USA). and cultured for 15 min at room temperature away from light. Finally, the distribution of cells in different apoptosis period was detected by Accuri C6 (BD Biosciences, SanDiego) as well.

### Wound-healing assay

We cultured stable transfected OS cells in six-well plates, which were then scratched by a 200-μL pipette tip to form two perpendicular wounds per well when the cells were grown to about 80% confluency. Subsequently, the wells were washed twice with PBS to wash off the unadherent cells. The images of wounds from the same position were photographed by an inverted microscope (Nikon) at 0 and 24 h, respectively. Finally, ImageJ software (NIH, Bethesda, MD, USA) was utilised to quantify the ratio of the area of wound healing within 24 h.

### Quantitative real-time PCR (RT-qPCR)

We extracted total RNA from OS cells under different treatment according to the instructions of RNeasy kit (Invitrogen). Then the extracted mRNAs were subjected to reverse transcription to cDNAs by using kits from the Accurate Biotechnology (Hunan, China). SYBR® Green Premix Pro Taq HS qPCR kit (Accurate Biotechnology, Hunan, China) assisted in quantifying obtained cDNAs, and an ABI Prism 7500 system (Applied Biosystems) was adopted to detect quantified cDNAs. The 2^-ΔΔCt^ method served for quantifing the fold change in gene expression. Every experiment was repeated three times independently. All primers are listed in Table 1.

**Table 1:** Primers used in the study.

Gene	Primers	
GAPDH	Forward:5′-ACAACTTTGGTATCGTGGAAGG-3′	Reverse:5′-GCCATCACGCCACAGTTTC-3′
FZD4	Forward: 5′-CCTCGGCTACAACGTGACC-3′	Reverse: 5′-TGCACATTGGCACATAAACAGA-3′
TNS1	Forward: 5′-GTACGTCACAGAGAGGATCATCG-3′	Reverse: 5′-GCAGGTAGTTGCCTCCATGTT-3′
STC2	Forward: 5′-GGGTGTGGCGTGTTTGAATG-3′	Reverse: 5′-TTTCCAGCGTTGTGCAGAAAA-3′
ZFP36	Forward: 5′-GACTGAGCTATGTCGGACCTT-3′	Reverse: 5′-GAGTTCCGTCTTGTATTTGGGG-3′
PRKAR1B	Forward: 5′-CAGGTCCTCAAAGACTGTATCGT-3′	Reverse: 5′-ATGGGAGTCCGACTGTGAGT-3′
CircPRKAR1B	Forward: 5′-CACGCTGAGGAAACGCAAGA-3′	Reverse: 5′-TTGTCTCCTTCATTCCCTTGCT-3′

### Western blotting analysis

OS cells were cultured in six-well plates with a suitable density. Cells were then exposed to different treatment for 48 h after complete adherence. Radioimmunoprecipitation assay (RIPA) lysis buffer (Sigma-Aldrich) that contained protease as well as phosphatase inhibitors was then used to extract the total proteins from each sample. In brief, 150 μL of mixed Ripa lysis buffer were added into each well of the six-well plates, which was then cracked on ice for 30 min. The liquid in each well was collected as completely as possible, and centrifuged at 4 °C for 15 min under the condition of 14000 *g*. Then the obtained supernatant was mixed with 5× loading buffer in proportion and boiled at 100 °C for 10 min. We stored the obtained protein samples at −20 °C. For Western blotting, we used SDS-PAGE (8–12%) to separate proteins and transferred them to 0.45-μm PVDF membranes (Bio-Rad, Hercules, CA, USA). The membranes were then blocked in 5% non-fat dry milk at room temperature for 1 h, after which the blocked membranes received one night of incubation with specific primary antibodies at 4°C. On the following day, TBST buffer was used to wash the incubated membrane for three times, and the washed membranes received 1 h of incubation with the corresponding secondary antibody at room temperature. Followed by another three times washing using TBST buffer, the protein band could be visualised by LAS-4000 Science Imaging System (Fujifilm, Tokyo, Japan). Image J was employed to analyse the results.

### Immunofluorescence staining

Firstly, PBS was used to wash cells for three times, and the washed cells were fixed in 4% paraformaldehyde for 20 min. Then, 0.5% Triton X-100 assisted in permeabilizing the fixed cells for 30 min before an 1-hour- blockade with 5% bovine serum albumin (BSA). After that, cells received one night of incubation by using specific primary antibodies at 4 °C. The corresponding secondary antibodies (Invitrogen) were used immunostained cells in the next day. Finally, PBS was used to wash cells for three times and DAPI (Beyotime Institute of Biotechnology, Shanghai,China) was adopted to stain the washed cells for ten mins to dye the nucleus. Image of immunofluorescence was further acquired from a fluorescence microscope (Olympus, Tokyo, Japan).

### Histology analysis and immunohistochemistry

The chondroma, osteosarcoma samples along with tumours and vital organs arrived from xenograft tumorigenesis models were preserved as mentioned above. The specimens were then subjected to section into a thickness of about 5 μm and stained with haematoxylin and eosin (H&E) for the subsequent histology analysis. As for immunohistochemistry, sections were stained using a histo-stain SABC kit (CWBIO, Shanghai) according to the instructions of the manufacturer. The specific primary antibody was used at a dilution of 1:100 in this study. Image-Pro Plus 6.0 (NIH, Bethesda, MD, USA) was then used to calculate the percentage of positive cells, which is determined by the proportion of cells that were positive for the marker in all cells. The percentage of positive cells in all cells. All histological analyses were independently reviewed in parallel by specialists from the pathology department of Sir Sun Shaw Hospital.

### Fluorescence in situ hybridisation (FISH)

Fluor 488-labelled circPRKAR1B probes as well as Fluor 555-labelled miR-361-3p probe were obtained from RiboBio. FISH analysis was determined with a FISH Kit (RiboBio) in terms of the manufacturer’s instructions. DAPI stained the cell nuclei. Images were further acquired from a fluorescence microscope (Olympus, Tokyo, Japan).

### Dual-luciferase reporter assay

Dual-luciferase came from Hanbio. 293 T cells were cotransfected with plasmid mixtures with or without the miR-361-3p mimics or negative control (NC) mimics with Lipofectamine RNAiMAX (Invitrogen) and cultured for 24 h. The activities of luciferase were measured following the manufacturer’s protocol.

### RNA immunoprecipitation (RIP)

RIP experiments were used to clarify whether circPRKAR1B bound to Ago2 and circPRKAR1B flanking sequence bound to EIF4A3. As our previous research reported [24], the Magna RIP RNA-Binding Protein Immunoprecipitation Kit (Millipore, Bedford, MA) was applied to RIP experiments.

### Antibodies

N-cadherin (abcam, ab76011) E-cadherin (CST, 3195), EIF4A3 (abcam, ab32485) Vimentin (huabio, M1412-1), FZD4 (abcolonal, A15113, A8161), β-Catenin (CST, #8480) Cyclin D1(CST, 55506), c-Myc (CST, 18583).

### Pull-down assay

Biotinylated-circPRKAR1B probe and antisense circPRKAR1B flanking RNA sequences came from RibiBio. The detailed steps are described in the previous research [25]. Silver staining together with WB confirmed the eluted proteins.

### Statistical analysis

SPSS 19.0 (SPSS, Chicago) served for data analyses. All quantitative data are in the form of mean ± SD. The two-tailed Student’s *t* test or one-way ANOVA followed by Tukey’s post hoc analyses helped to analyse the statistical significance, unless noted otherwise. *P* values < 0.05 exhibited statistical significance.

## Results

### circPRKAR1B is overexpressed in OS tissues and cell lines

**Fig. 1:**
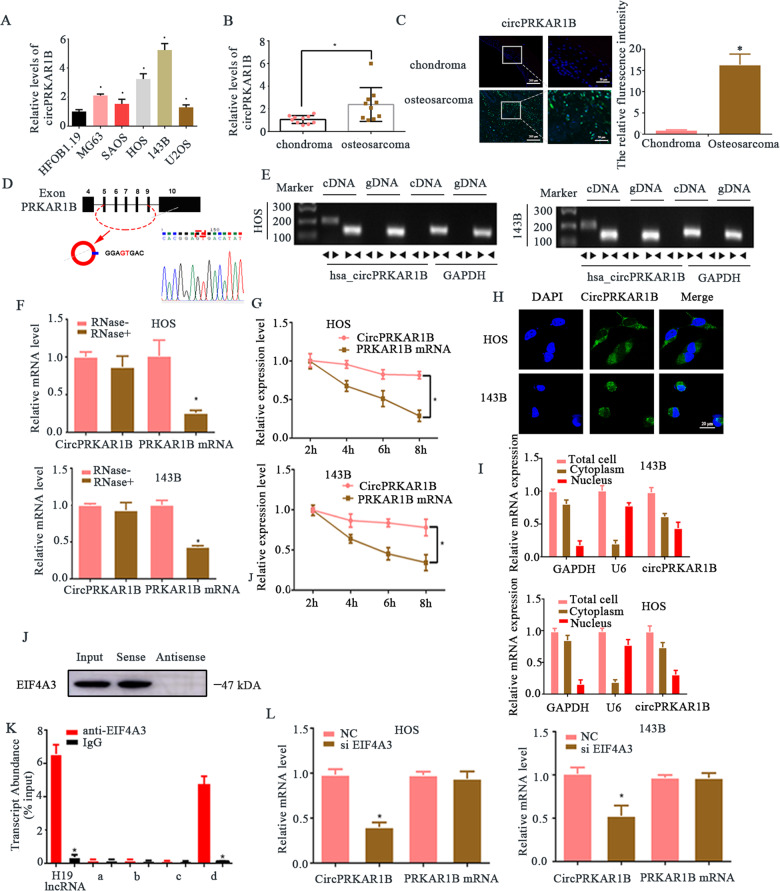
Validation and expression of circPRKAR1B in OS tissues and cells. **A** Expression of circPRKAR1B in OS cell lines compared with control cells (HFOB1.19 cells). **B** The level of circPRKAR1B was higher in human OS tissue samples than chondroma samples. **C** FISH showed that circPRKAR1B expression was higher in OS tissues than chondroma tissues. **D** Schematic diagram illustrating that exons 5–9 of PRKAR1B form circPRKAR1B. **E** Agarose gel electrophoresis showing the presence of circPRKAR1B in HOS and 143B cells by RT-PCR. Divergent primers amplified circPRKAR1B from cDNA but not from genomic DNA. GAPDH was used as a negative control. **F** The expression of circPRKAR1B and PRKAR1B mRNA in HOS and 143B cells treated with or without RNase R, as determined by RT-qPCR. **G** The levels of circPRKAR1B and PRKAR1B in HOS and 143B cells treated with actinomycin D at the indicated time points were measured by RT-qPCR. **H** FISH revealed that circPRKAR1B was predominantly localised in the cytoplasm. Nuclei were stained with DAPI, and circPRKAR1B probes were labelled with Alexa Fluor 488. **I** qRT-PCR was performed to determine the main localisation of circPRKAR1B in OS cells. **J** Western blotting were used to demonstrate the interaction between EIF4A3 and the circPRKAR1B downstream region. **K** RIP was performed to verify that eIF4A3 binds PRKAR1B mRNA. H19 lncRNA was used as the positive control, and RT-qPCR was used to measure transcript levels relative to the input. **L** HOS and 143B cells were transfected with control or a plasmid to knock down eIF4A3, and circPRKAR1B expression was measured by RT-qPCR. Scale bar = 20 μm. The data represent the mean ± SD of three independent experiments (*P < 0.05).

CircPRKAR1B level was firstly determined in the tissues and cells of OS. In terms of the circPRKAR1B content, RT-qPCR results indicated that multiple OS cell lines (MG63, SAOS, HOS, 143B and U2OS), especially 143B and HOS cells, obtained a much higher value than the HFOB1.19 (Fig. 1A). CircPRKAR1B was upregulated in OS tissues compared with chondroma tissues (Fig. 1B). A notable increase in circPRKAR1B expression was detected in OS tissues by fluorescence in situ hybridisation (FISH) staining using a specific circPRKAR1B probe indicated (Fig. 1C). By comparing the sequences of PRKAR1B mRNA and circPRKAR1B, we found that circPRKAR1B is looped and contains exons 5 to 9 of its parental gene. Furthermore, Sanger sequencing assisted in verifying the head-to-tail splicing structure (Fig. 1D). Meanwhile, we designed convergent and divergent primers for amplifying PRKAR1B mRNA and circPRKAR1B. Electrophoresis was used to detect cDNA and genomic DNA (gDNA) in HOS and 143B cells. CircPRKAR1B was amplified by divergent primers from cDNA, but not gDNA (Fig. 1E). In contrast, PRKAR1B mRNA was only amplified by convergent primers and could be detected in cDNA as well as gDNA. To test the stability of circRNA, we performed an RNase R assay and RT-qPCR. CircPRKAR1B exhibited a remarkable resistance to RNase R digestion (Fig. 1F) and Actinomycin D treatment (Fig. 1G). RNA FISH and RT-qPCR analysis revealed that circPRKAR1B was mainly localised in the cytoplasm (Fig. 1H, I). Taken together, these results revealed that circPRKAR1B is upregulated in OS and, thus, may contribute to OS progression.

### EIF4A3 promotes circPRKAR1B expression

We used circinteractome tool to predict the regulators of circPRK1R1B and found EIF4A3 could bind to the downstream of circPRK1R1B. We then performed RNA pull-down assay of circPRKAR1B flanking sequence and confirmed that EIF4A3 was the most enriched protein at circPRKAR1B flanking sequences (Fig. 1J). We then used circinteractome tool to predict the binding regions of cirPRKAR1B and EIF4A3 (Fig. S1). We truncated the full-length, upstream and downstream of circPRKAR1B into four segments (a–d). In line with the prediction, RNA immunoprecipitation (RIP) assays (Fig. 1K) indicated that EIF4A3 binds to one downstream flanking sequence (named d) but not the upstream site or other sites (a–c). Knockdown of EIF4A3 inhibited the biogenesis of circPRKAR1B in HOS and 143B cell lines, while mPRKAR1B did not exhibit markedly changes (Fig. 1L). Cumulatively, EIF4A3 promotes the expression of circPRKAR1B by binding to its downstream flanking sequences.

### The role of circPRKAR1B in the proliferation of OS cells and their adoption of an EMT-like phenotype

**Fig. 2:**
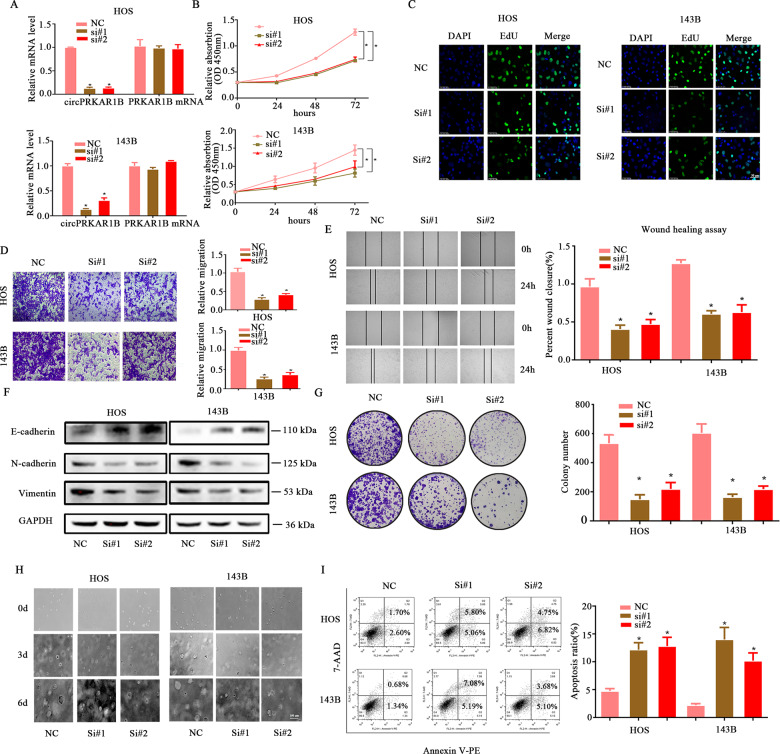
circPRKAR1B knockdown inhibited the migration and proliferation of OS cells. **A** HOS and 143B cells were stably transfected with circPRKAR1B siRNA or NC, and the mRNA expression levels of circPRKAR1B and PRKAR1B were measured by RT-qPCR. **B** The proliferation of HOS and 143B cells transfected with sh-circPRKAR1B was measured by the CCK-8 assay. **C** CircPRKAR1B plays a role in tumour cell proliferation, as determined by the EdU assay. Nuclei were stained with DAPI, and the combination of EdU and DAPI indicated cells in S phase. **D** The migration abilities of HOS and 143B cells transfected with si-circPRKAR1B or NC were evaluated by Transwell migration assays. **E** The effect of si-circPRKAR1B on migration was evaluated by the wound-healing assay in HOS and 143B cells. **F** The protein expression of N-cadherin, E-cadherin and Vimentin was measured by Western blot analysis in both HOS and 143B cells transfected with si-circPRKAR1B. **G** SiRNA-mediated circPRKAR1B knockdown significantly suppressed cell growth in the colony formation assay. **H** circPRKAR1B knockdown inhibited the anchorage-independent colony-forming abilities of both HOS and 143B cells. **I** HOS and 143B cells were transfected with si-circPRKAR1B and then subjected to Annexin V-PE/7AAD staining. The percentage of apoptotic cells is shown. The data represent the mean ± SD (*n* = 3) (*P < 0.05).

Two circPRKAR1B siRNAs were used to stably silence circPRKAR1B expression and assess the function of circPRKAR1B in OS. CircPRKAR1B was knocked down by siRNAs, but the level of PRKAR1B mRNA was not affected (Fig. 2A). In addition, cell viability was further evaluated by the CCK-8 and EdU assays (Fig. 2B, C), which indicated that circPRKAR1B knockdown impaired proliferation. Similarly, migration ability was decreased by depletion of circPRKAR1B (Fig. 2D), which was also tested by the wound-healing assay (Fig. 2E).

It is well known that mesenchymal migration of OS cells regulated by EMT is a key process in regulating metastasis of OS. Given circPRKAR1B modulation affects migration of OS cells, we explore whether circPRKAR1B could regulate EMT by detecting N-cadherin, E-cadherin and vimentin. As expected, OS cells transfected with shRNA displayed a pronounced decrease in the expression of the mesenchymal markers N-cadherin and vimentin and increased expression of the epithelial marker E-cadherin (Fig. 2F). Silencing of circPRKAR1B also significantly impaired the colony-forming ability of OS cells (Fig. 2G). The soft agar assay results also showed that the colony-forming ability of OS cells was inhibited upon circPRKAR1B knockdown (Fig. 2H). Knockdown of circPRKAR1B induced apoptosis of OS cells (Fig. 2I). Hence, these results prove that circPRKAR1B plays roles in the proliferation of OS cells and their adoption of an EMT-like phenotype in vitro.

We then performed gain-of-function experiments (Fig. s2A) and found that overexpression of circPRKAR1B cause OS cells to switch from an epithelial phenotype to a mesenchymal phenotype. Cells transfected with circPRKAR1B increased circPRKAR1B expression but did not affect host PRKAR1B level (Fig. s2A, B). Besides, the abilities of proliferation, migration and colony-forming in OS cells were enhanced by circPRKAR1B overexpression (Fig. s2 B–F).

### The role of circPRKAR1B as a sponge for miR-361-3p, which inhibits the degree of OS malignancy

**Fig. 3:**
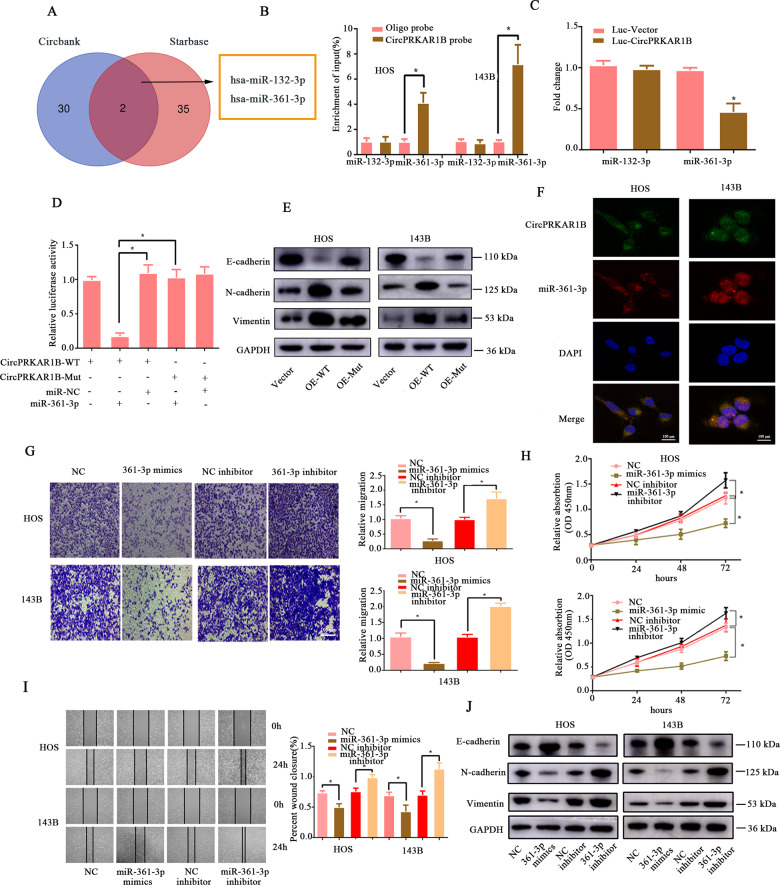
CircPRKAR1B acts as a sponge for miR-203a-3p, which suppresses OS cell proliferation, migration, and EMT. **A** Schematic illustration exhibiting the overlapping of the target miRNAs of circTADA2A predicted by circBank and starBase. **B** Lysates prepared from HOS and 143B cells stably transfected with circPRKAR1B or vector were subjected to RNA pull-down assays and were analysed by RT-qPCR. The relative levels of circPRKAR1B pulled down by the circPRKAR1B probe were normalised to the level of circPRKAR1B pulled down by an oligo probe. **C** Relative luciferase activity of the circPRKAR1B luciferase reporter after cotransfection of different candidate miRNA mimics into HEK-293T cells. **D** HEK-293T cells were co-transfected with miR-361-3p mimics or NC and the wild-type or mutant circPRKAR1B luciferase reporter and subjected to the luciferase assay. **E** Western blot analyses of EMT-associated proteins after the overexpression of circPRKAR1B or its mutant. **F** FISH revealed colocalization between miR-361-3p and circPRKAR1B in HOS and 143B cells. CircPRKAR1B probes were labelled with Alexa Fluor 488. MiR-361-3p probes were labelled with Alexa Fluor 555. Nuclei were stained with DAPI; **G** HOS and 143B cells were transfected with miR-361-3p mimics or a miR-361-3p inhibitor, and cell migration was evaluated after 48 h. **H** Downregulation of miR-361-3p expression stimulated cell growth, and overexpression of miR-361-3p suppressed cell growth in HOS and 143B cells. **I** Migration ability of transfected PTC cells was evaluated by the wound-healing assay. **J** The protein expression of N-cadherin, E-cadherin, and Vimentin was measured by Western blot analysis in both HOS and 143B cells transfected with miR361-3p-mimics or a miR-361-3p inhibitor. The data represent the mean ± SD of three independent experiments (*P < 0.05).

The underneath mechanism by which circPRKAR1B regulated OS was needed to discover. As reported [26, 27], cytoplasm-localised circRNAs could act as miRNA sponges. To investigate the miRNA-binding ability of circPRKAR1B, we conducted an AGO2 RIP assay and observed that endogenous circPRKAR1B was specifically enriched based on RT-qPCR analysis, which indicated that circPRKAR1B participates in translational regulation by acting as ceRNAs (Fig. s3A). Thus, we screened all miRNAs containing the binding sites of circPRKAR1B and found miR-132-3p and miR-361-3p were suitable according to two bioinformatics databases (circBank and starBase) (Fig. 3A). However, further RNA pulldown assay revealed that the interreaction relation exists in circPRKAR1B with miR-361-3p but not with miR-132-3p (Fig. 3B). Subsequently, dual-luciferase reporter assay confirmed the miR-361-3p interacts with circPRKAR1B (Fig. 3C). Starbase online database showed the binding sequence between miR-361-3p and circPRKAR1B (Fig. s3 B). We then constructed a luciferase reporter gene vector containing either the WT or mutant (MUT) circPRKAR1B and then cotransfected miR-361-3p mimics with the luciferase reporter gene vectors into 293 T cells. Compared with the other groups, the miR-361-3p mimic reduced the luciferase reporter activity in 293 T cells transfected with the WT circPRKAR1B (Fig. 3D). Overexpression of circPRKAR1B-MUT rescued the effection on EMT-related proteins in circPRKAR1-WT determined by Western blot analyses (Fig. 3E). In addition, double FISH revealed the colocalization of circPRKAR1B and miR361-3p by fluorescence confocal microscopy (Fig. 3F).

Subsequently, we further assessed the effects of miR-361-3p in OS cells line. MiR-361-3p plays a role in inhibiting oncogenesis in numerous neoplastic diseases, but its function in OS tissues has never been reported [28, 29]. According to the RT-qPCR results, miR-361-3p expression was lower in osteosarcoma tissues than in chondroma tissues and was downregulated in OS cells compared with hFob1.19 cells (Fig. s3 C, D). OS cells were transfected with miR-361-3p mimics or a miR-361-3p-inhibitor to explore the function of miR-361-3p in OS. The migration (Fig. 3G), colony formation (Fig. s2 E) and wound-healing assays (Fig. 3I) showed miR-361-3p mimics suppressed migration and proliferation, whereas suppression of miR-361-3p had the opposite effects. Our results showed that that miR-361-3p overexpression significantly decreased cell viability in OS cells, while the miR-361-3p inhibitor had the opposite effect (Fig. 3H). Notably, miR-361-3p mimics suppressed the expression of mesenchymal markers (N-cadherin and Vimentin) and led to the upregulation of the expression of an epithelial marker (E-cadherin), which indicated that miR-361-3p could inhibit the EMT transition of OS cells (Fig. 3J). Collectively, the results of these experiments indicated that circPRKAR1B acted as a sponge of miR361-3p in OS cells.

### miR-361-3p directly targets FZD4 and is involved in Wnt/β-catenin signalling in OS

**Fig. 4:**
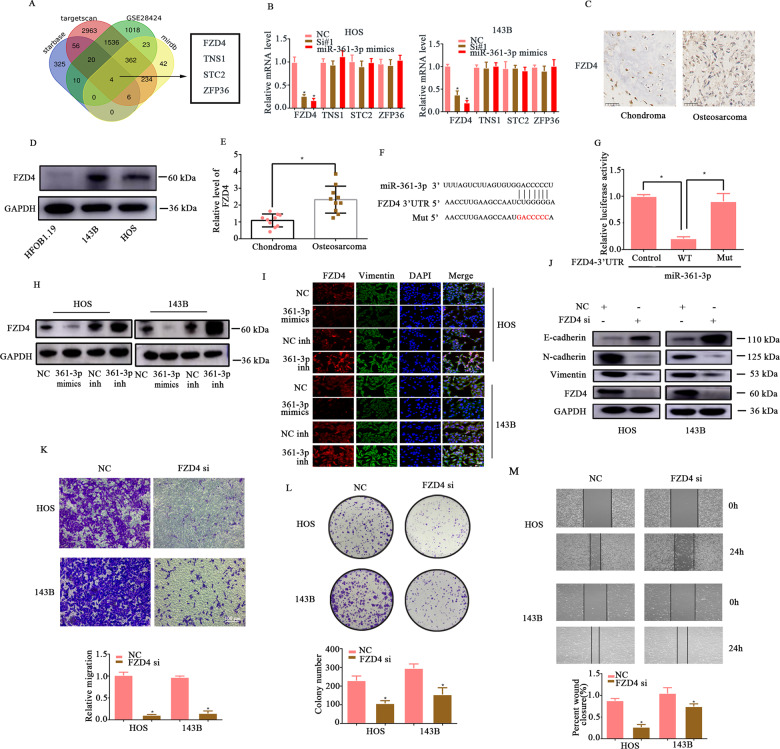
FZD4 is a direct target of miR361-3p. **A** Schematic illustration exhibiting the overlapping of the target genes of miR-361-3p predicted by starBase, TargetScan, GSE28424 and miRDB. **B** Four upregulated genes were subjected to RT-qPCR in HOS and 143B cells transfected with either Si1-circPRKAR1B or miR-361-3p mimics. **C** Immunohistochemistry (IHC) results showing that FZD4 expression was upregulated in OS tissues compared with chondroma tissues. **D** The protein expression of FZD4 measured detected by Western blot analysis in both HOS, 143B and HFOB1.19 cells. **E** RT-qPCR demonstrated a difference in the expression of FZD4 at the mRNA level between osteosarcoma and chondroma tissues (*n* = 10). **F** Schematic illustration of the complementary sequence between miR-361-3p and FZD4. Mutated nucleotides in the FZD4 3′UTR are shown in lowercase letters. **G** HEK-293T cells were transfected with miR-361-3p mimics or NC and the wild-type or mutated FZD4 3′-UTR reporter and subjected to the luciferase assay. OS cells were stably transfected with miR-296-3p mimics and a miR-296-3p inhibitor. FZD4 expression was measured by Western blotting (**H**) and IF (**I**) at the protein level. **J** The protein levels of β-catenin, cyclin D1, C-myc, E-cadherin, N-cadherin, vimentin and FZD4 in OS cells when FZD4 was knocked down. **K** The cell migration abilities of HOS and 143B cells transfected with NC or si-FZD4 were evaluated by Transwell migration assays. **L** Colony formation assay showing changes in the proliferative capacity of OS cells stably transfected with NC or si-FZD4. **M** Representative images showing the effect of FZD4 knockdown on migration ability, as demonstrated by the wound-healing assay. The data represent the mean ± SD (*n* = 3) (*P < 0.05).

**Fig. 5:**
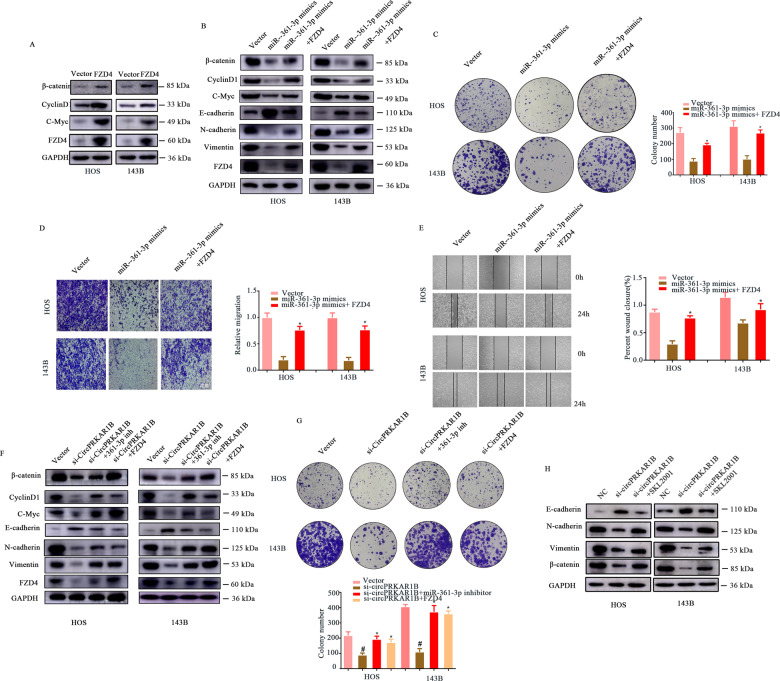
circPRKAR1B promotes OS progression via the miR-361-3p/FZD4-Wnt/β-catenin axis. **A** The protein levels of β-catenin, cyclin D1, C-myc and FZD4 in OS cells trasfected with FZD4 plasmid. **B** The protein levels of β-catenin, cyclin D1, C-myc, E-cadherin, N-cadherin, vimentin and FZD4 in OS cells transfected with miR-361-3p with or without FZD4 overexpression. **C** The reversal effect of FZD4 overexpression on the colony-forming ability of OS cells transfected with miR-361-3p mimics. **D** The enhancement of the migration ability of HOS and 143B cells was partially abrogated by co-transfection with any of the miR-361-3p mimics or FZD4 overexpression. **E** Representative images showing the reversal of migration ability upon FZD4 upregulation by the wound-healing assay. **F** WB revealed the reversal effect of the miR-361-3p inhibitor or FZD4 overexpression on EMT and the Wnt/β-catenin signalling pathway in OS cells with circPRKAR1B knockdown. **G** The colony formation assay showed changes in the proliferation capacity of OS cells stably expressing si-circPRKAR1B transfected with or without the miR-361-3p inhibitor or FZD4 overexpression plasmid. **H** WB revealed the rescue effect of the β-catenin agonist (SKL2001) on EMT in OS cells with si-circPRKAR1B. The data represent the mean ± SD (*n* = 3) (*P < 0.05).

To further explore the downstream targets of the circPRKAR1B/miR-361-3p axis, the prediction results of the TargetScan, starBase, GSE28424 and MiRdb were compared, which led to the identification of four possible mRNAs, namely, FZD4, TNS1, STC2 and ZFP36 (Fig. 4A). To determine the target gene of miR-361-3p, RT-qPCR was performed and the results showed that the expression of only one candidate (FZD4) was downregulated by the transfection of both si-circPRKAR1B and miR-361-3p mimics in OS cells (Fig. 4B). Next, immunohistochemical staining showed that FZD4 expression was strongly increased in OS tissues compared with chondroma tissues (Fig. 4C). Consistent with the IHC results, RT-qPCR and Western blotting revealed high expression of FZD4 in OS tissues and cells (Fig. 4D, E). As illustrated in Fig. 4G, the FZD4 3′ UTR contains sequences that are complementary to those of miR-361-3p (Fig. 4F). To further determine whether FZD4 harbours an miR-361-3p binding domain, we constructed a luciferase reporter gene vector containing either the WT or mutant (MUT) FZD4 and then cotransfected miR-361-3p mimics with the luciferase reporter gene vectors into 293T cells. It was found that miR-361-3p mimics markedly reduced the luciferase activity in FZD4 WT-transfected cells, and this reduction was abolished by transfection with FZD4 Mut, which indicated the binding region of FZD4 and miR-361-3p (Fig. 4G). Additionally, FZD4 expression was markedly downregulated after transfection of miR-361-3p mimics and was notably upregulated by miR-361-3p inhibitor (Fig. 4I, J), indicating that miR-361-3p inversely regulated FZD4 at the protein level. Next, we further assessed the function of FZD4 in OS cell lines. FZD4 was knocked down by transfection with siRNA (Fig. 5K). Knockdown of FZD4 resulted in the inhibition of migration and colony forming, which were assessed by transwell migration assays, wound healing assays and colony forming assays (Fig. 4L–N). Taken together, these data suggested that miR-361-3p targeted FZD4.

### circPRKAR1B facilitates OS progression through the FZD4/Wnt/β-catenin axis

Given the binding relationship between miR-361-3p and FZD4, whether miR-361-3p regulated OS process by targeting FZD4 was continued to be explored. FZD4 has been reported to promote Wnt/β-catenin signalling and markedly affect numerous neoplastic diseases [30, 31], but there are few reports on the function of FZD4 in osteosarcoma. Results firstly presented FZD4 protein expression and Wnt/β-catenin signalling related proteins were dramatically upregulated in 143B and HOS cells transfected with FZD4 overexpression plasmid (Fig. 5A). To verify whether miR-361-3p inhibits cell migration and EMT in OS via the FZD4/Wnt/β-catenin axis, 143B and HOS cells were cotransfected with miR-361-3p mimics and a FZD4 overexpression (OE) plasmid. It was found that the levels of β-catenin, cyclin D1, C-myc, Vimentin and N-cadherin were significantly increased, while E-cadherin expression was reduced in cells cotransfected with miR-361-3p mimics and the FZD4-OE plasmid compared with those transfected with miR-361-3p mimics alone (Fig. 5B). The colony-forming ability of OS cells was rescued by cotransfection of miR-361-3p mimics and the FZD4-OE plasmid (Fig. 5C). Moreover, the transwell migration and wound healing assays showed that OS cells cotransfected with miR-361-3p mimics and the FZD4-OE plasmid exhibited increased migration ability compared with those transfected with the miR-361-3p mimic along (Fig. 5D, E). Consistently, the soft agar assay and EdU assay showed that the colony-forming ability of OS cells was rescued upon cotransfection with FZD4 and the miR-361-3p mimics (Fig. s4A, B). Next, we explored whether circPRKAR1B was involved in activating Wnt/β-catenin signalling through the miR-361-3p/FZD4 axis. The results showed that knockdown of circPRKAR1B decreased the expression of FZD4, β-catenin, cyclin D1, C-myc, N-cadherin and Vimentin, and increased the level of E-cadherin, but these changes were prevented by transfection of both the miR-361-3p inhibitor and the FZD4-OE plasmid (Fig. 5F). The Transwell, wound healing and colony forming assays confirmed the rescue effects of the miR-361-3p inhibitor and FZD4-OE plasmid (Fig. 5G and Fig. s4 C, D). We overexpressed beta catenin (SKL2001, 30 μM) and found that it can partially rescue the onco-suppressor activities of the silencing of circPRKAR1B (Fig. 5H). In summary, these results demonstrated that circPRKAR1B promotes OS progression via the FZD4/Wnt/β-catenin axis.

### Knockdown of circPRKAR1B inhibits OS growth and metastasis in vivo

**Fig. 6:**
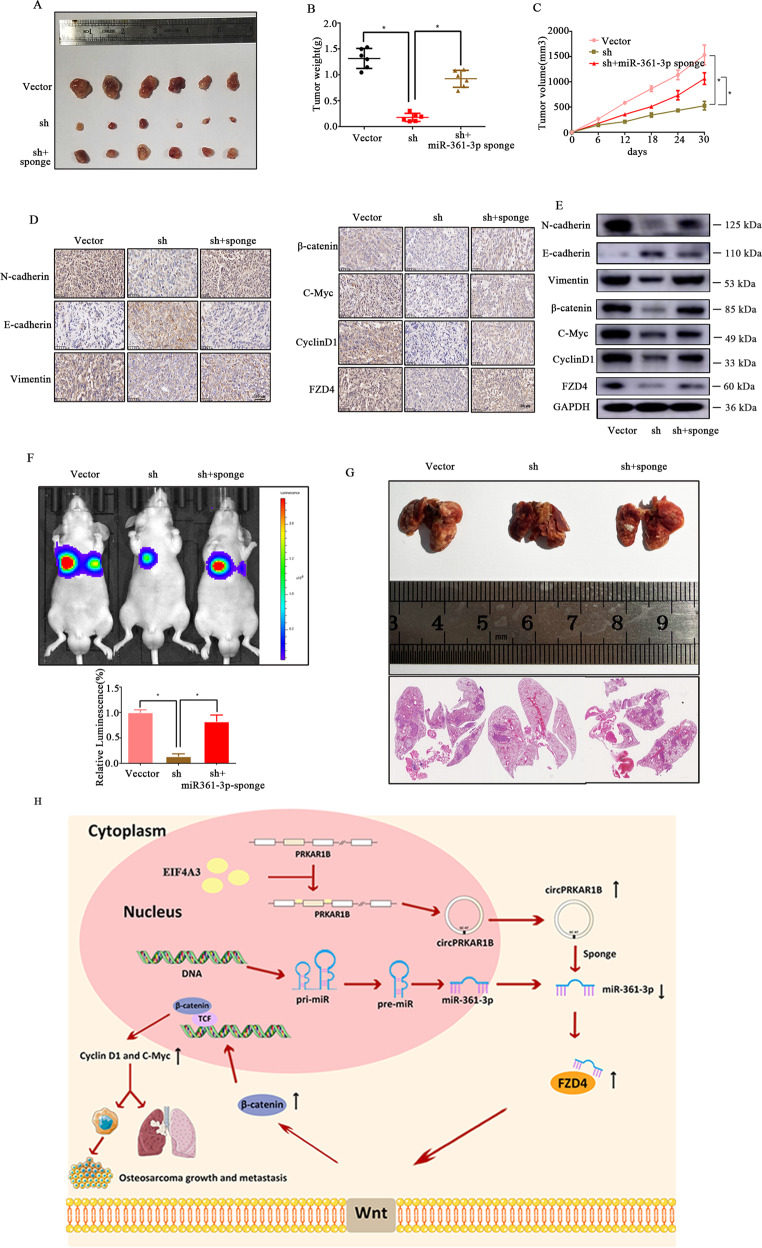
CircPRKAR1B functions as a miR-361-3p sponge to promote tumorigenesis in vivo. **A** Nude mice were subcutaneously injected with 5 × 10^6^ cells stably transfected with the control or circPRKAR1B shRNA or cotransfected with circPRKAR1B shRNA and miR-361-3p sponge. After 30 days, the tumours were dissected and photographed. **B** miR-361-3p knockdown promoted the growth of tumours derived from circPRKAR1B knockdown cells, and the average tumour weight in each group at the end of the experiment (day 30) is shown. The data represent the mean ± SEM (*n* = 6) (*p < 0.05). **C** Tumour volumes were recorded every six days after mice were injected with stable OS cells. The data represent the mean ± SEM (*n* = 6) (*p < 0.05). N-cadherin, E-cadherin, vimentin, β-catenin, C-myc, cyclin D1 and FZD4 expression levels were determined by immunohistochemistry (**D**) and WB (**E**). Scale bars, 100 mm. **F** Lung metastasis of mice injected with different stably transfected 143B cells via the tail vein was detected using an in vivo bioluminescence imaging system. Representative images and a histogram are shown (*n* = 6 each group). **G** Histological analysis of lung tissues by haematoxylin and eosin staining. **H** Schematic illustration of the EIF4A3-induced circPRKAR1B/miR-361-3p/FZD4 axis. The data are the mean ± SD of three independent experiments (**D**–**F**) (*P < 0.05).

To determine the roles played by circPRKAR1B and miR-361-3p in vivo, subcutaneous xenograft tumour and lung metastasis models were established. 143B cells (labelled with a luminescent dye) were stably transfected with vector or sh-circPRKAR1B or cotransfected with sh-circPRKAR1B. Nude mice were injected with a miR-361-3p sponge. The tumours derived from sh-circPRKAR1B were smaller than those derived from the vector, while transfected with the miR-361-3p sponge partially prevented this difference in tumour size (Fig. 6A). Final tumour weights and tumour volumes in these groups revealed similar findings (Fig. 6B–C). In addition, circPRKAR1B knockdown and transfection of the miR-361-3p sponge led to a decrease and increase in mesenchymal phenotype, respectively. Moreover, IHC and Western blotting showed that FZD4, C-myc, Cyclin D1, and β-catenin expression was inhibited by sh-circPRKAR1B and rescued by the miR-361-3p sponge, which was consistent with our in vitro results (Fig. 6D, E). OS tends to metastasise, and bioluminescence imaging showed that the formation of pulmonary metastasis tumours by 143B cells was markedly suppressed in the sh-circPRKAR1B group compared with the vector group and that the miR-361-3p sponge reversed this effect (Fig. 6F). Moreover, circPRKAR1B knockdown notably decreased the number of metastatic nodules in the lung (Fig. 6G). In summary, circPRKAR1B may crucially affect tumour proliferation and lung metastasis in OS through EIF4A3-induced circPRKAR1B/miR-361-3p/FZD4 axis (Fig. 6H).

### Overexpression of circPRKAR1B suppresses the sensitivity of OS cells to Cisplatin (CDDP)

**Fig. 7:**
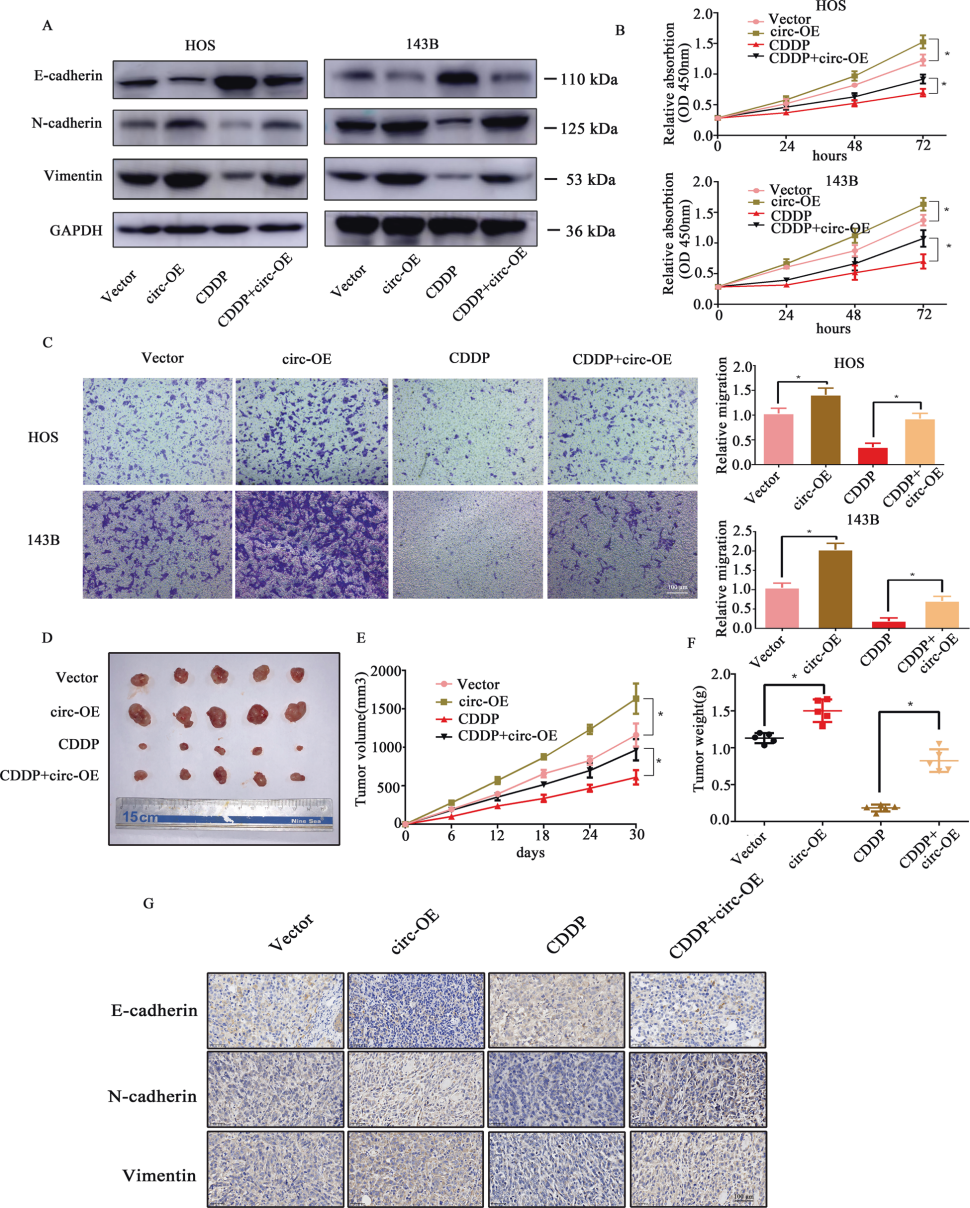
Overexpression of circPRKAR1B suppresses the sensitivity of OS cells to CDDP. **A** Protein levels of molecules related to EMT in OS cells treated as indicated. **B** CCK8 assay was used to analyse the growth capacity of OS cells treated as indicated. **C** The migration ability of HOS and 143B cells treated as indicated. **D** Images of a tumour xenograft model (*n* = 5). **E** Statistical analysis of tumour volume among the four indicated groups on the indicated days. **F** Statistical analysis of tumour weight among the four indicated groups. **G** Immunohistochemical analyses of xenograft tumours. IHC staining was performed using antibodies against E-cadherin, N-cadherin and Vimentin. The error bars represent the SD of three independent experiments (*P < 0.05).

The development of chemotherapeutic drug resistance worsens the prognosis of patients with osteosarcoma, and therefore, it is important to assess the underline mechanism of the sensitivity of OS cells to chemotherapy drugs. After 24 h of transfection, the cells were treated with 1 μM CDDP. Figure 7A illustrates that circPRKAR1B overexpression reversed the inhibition of the EMT phenotype in OS cells caused by CDDP. The CCK8 assay showed that cell proliferation ability was increased in circPRKAR1B cells after treatment with CDDP (Fig. 7B). The transwell migration assay showed that the migration rate was decreased after treatment with CDDP. Interestingly, this effect was abrogated when circPRKAR1B expression was upregulated (Fig. 7C). In vivo experiments, from the 7th day of inoculation, CDDP (8 mg/kg) was intraperitoneally administered to the mice. We found that tumour sizes and weights were clearly decreased after treatment with CDDP in the vector group. Consistent with the in vitro experiments, circPRKAR1B overexpression rescued this effect (Fig. 7D–F). Moreover, IHC showed that the expression of N-cadherin and vimentin was increased and that the expression of E-cadherin was downregulated after treatment with CDDP (Fig. 7G). These data indicated that overexpression of circPRKAR1B suppresses the sensitivity of OS cells to CDDP.

## Discussion

OS is a complex disease that is highly malignant and metastatic in children and adolescents [32]. Advanced surgery combined with chemotherapeutic agents, such as cisplatin, doxorubicin, and methotrexate, is often applied to treat OS [33, 34]. However, the survival rate of OS is still unsatisfactory due to drug resistance or potential distant metastasis [3]. Therefore, new treatments need to be identified, and the mechanisms underlying OS need to be explored to improve OS patient prognosis.

CircRNAs, noncoding RNAs that are tissue specific and highly conserved in mammals, have attracted much attention due to their features and functions in recent years [35, 36]. Emerging evidence has suggested that circRNAs have multiple functions, including acting as miRNA sponges, undergoing translation, regulating the transcription of their parental gene, and adsorbing RNA-binding proteins [37, 38]. Our early studies showed that circFAT1 serves as a sponge specific for miR-375, which enhances YAP1 expression and accelerates OS progression [39]; that circTADA2A promotes OS cell apoptosis, migration, and invasion [25]; and that circMYO10 accelerates OS progression and metastasis [40]. In the present study, we found that circPRKAR1B, which is derived from exons 5 to 9 of PRKAR1B, is clearly overexpressed in OS tissues and cell lines and can act as a sponge for miR-361-3p to regulate FZD4 expression. CircPRKAR1B knockdown suppressed migration, proliferation, EMT and metastasis in vitro and in vivo, demonstrating that it plays an antioncogenic role in OS progression. Based on the competing endogenous RNA hypothesis, circRNAs can communicate with miRNAs and regulate miRNA levels by competing with endogenous miRNA sponges to modulate the level of oncogenes [41]. Consistent with this phenomenon, our bioinformatics analysis, RNA pull-down assay and dual luciferase reporter assays showed direct binding between circPRKAR1B and miR-361-3p. These data demonstrated that circPRKAR1B might function as a ceRNA to accelerate the oncogenesis and progression of OS.

Widely accepted as a tumour inhibitor, miR-361-3p was reported to suppress the Wnt signalling pathway, which could inhibit distant metastasis of tumours [42, 43] and has been found to be expressed in gastric cancer [44], hepatocellular carcinoma [45] and cervical cancer [46]. However, to our knowledge, there has been no research related to miR-361-3p in bone neoplasms. This study revealed that circPRKAR1B can bind to miR-361-3p and affect FZD4 expression, thereby promoting OS progression. FZD4 is associated with the activation of the Wnt/β-catenin signalling pathway in multiple diseases [47, 48]. Notably, FZD4 was overexpressed in OS tissues and cell lines, and silencing FZD4 led to a significant decrease in β-catenin expression in HOS and 143B cells and was able to alleviate OS. In the Wnt/β-catenin signalling pathway, the β-catenin protein markedly promotes the expression of oncogene. It has been reported that when the Wnt/β-catenin signalling pathway is activated in the neural crest, it can increase the expression of cyclin D1 and C-myc [49], which is largely consistent with the data of our study. A recent study concluded that targeting the Wnt/β-catenin pathway can inhibit EMT and distant pulmonary metastasis in OS [50]. Our current results revealed that when FZD4 was knocked down, the expression of mesenchymal markers (N-cadherin and vimentin), cyclin D1 and C-myc was downregulated, while that of an epithelial marker (E-cadherin) was upregulated. C-myc accelerates osteosarcoma growth and metastasis, whereas knockdown of C-myc results in the opposite outcome. Furthermore, cyclin D1 is related to cell proliferation, migration, EMT and chemoresistance in several types of cancers [51]. Moreover, overexpression of FZD4 abolished the impact of miR-361-3p and the effect of circPRKAR1B knockdown. Thus, circPRKAR1B promotes OS progression and metastasis through modulating the miR-361-3p/FZD4/Wnt/β-catenin axis.

Previous studies have identified EIF4A3 as a core contributor to mRNA splicing [52]. As revealed in this study, EIF4A3 can combine with the downstream region of circPRKAR1B pre-mRNA and regulate the expression of circPRKAR1B pre-mRNA. Hence, EIF4A3 modulates circPRKAR1B formation. CDDP combined with advanced surgery is widely used for the treatment of OS, but as drug resistance increases, the prognosis of patients becomes more unfavourable. Numerous noncoding RNAs, such as circRNAs and miRNAs, have been shown to be involved in the development of drug resistance in OS [53, 54]. According to in vitro and in vivo studies, overexpression of circPRKAR1B suppressed the resistance of OS cells to CDDP.

## Conclusions

In conclusion, our research established a previously unknown function for circPRKAR1B in OS. Because circPRKAR1B affects cell proliferation, migration, and EMT, circPRKAR1B promotes oncogenesis and metastasis. Molecules related to the EIF4A3-induced circPRKAR1B/miR-361-3p/FZD4 axis, as a ceRNA network, have the potential to be developed as diagnostic biomarkers and targets for chemotherapy resistance in OS.

## Supplementary information

SUPPLEMENTAL MATERIAL

aj-checklist

## Data Availability

The data of this study are available from the first author and corresponding author upon reasonable request.
