# Supplementary material for: EIF4A3-induced circular RNA PRKAR1B promotes osteosarcoma progression by miR-361-3p-mediated induction of FZD4 expression
Source: Cell Death Dis. 2021 Oct 29;12(11):1025. doi: 10.1038/s41419-021-04339-7 (PMC8556261; doi:10.1038/s41419-021-04339-7)
Supplement: Supplementary file 1 — SUPPLEMENTAL MATERIAL [file 41419_2021_4339_MOESM1_ESM.docx]

**Supplemental Information**

**EIF4A3-induced circular RNA** **PRKAR1B promotes osteosarcoma progression by** **miR-361-3p-mediated induction of FZD4 expression**

Zhen-hua Feng^1,2 *^, Lin Zheng ^1,2 *^, Teng Yao ^1,2 *^, Si-yue Tao^1,2^, Xiao-an Wei^1,2^, Ze-yu Zheng^1,2^, Bing-jie Zheng^1,2^, Xu-yang Zhang^1,2^, Bao Huang^1,2^, Jun-hui Liu^1,2^, Zhi Shan^1,2^, Yi-lei Chen^1,2^, Pu-tao Yuan^1,2^, Cheng-gui Wang^3^, Jian Chen^1,2^, Shu-ying Shen^1,2#^, Feng-dong Zhao^1,2#^

1. Department of Orthopaedic Surgery, Sir Run Run Shaw Hospital, Zhejiang University School of Medicine, Hangzhou, China

2. Key Laboratory of Musculoskeletal System Degeneration and Regeneration Translational Research of Zhejiang Province, Hangzhou, China

3. Department of Orthopedics, 2nd Affiliated Hospital, School of Medicine, Zhejiang University, Hangzhou, China.

**Supplemental figures**

**
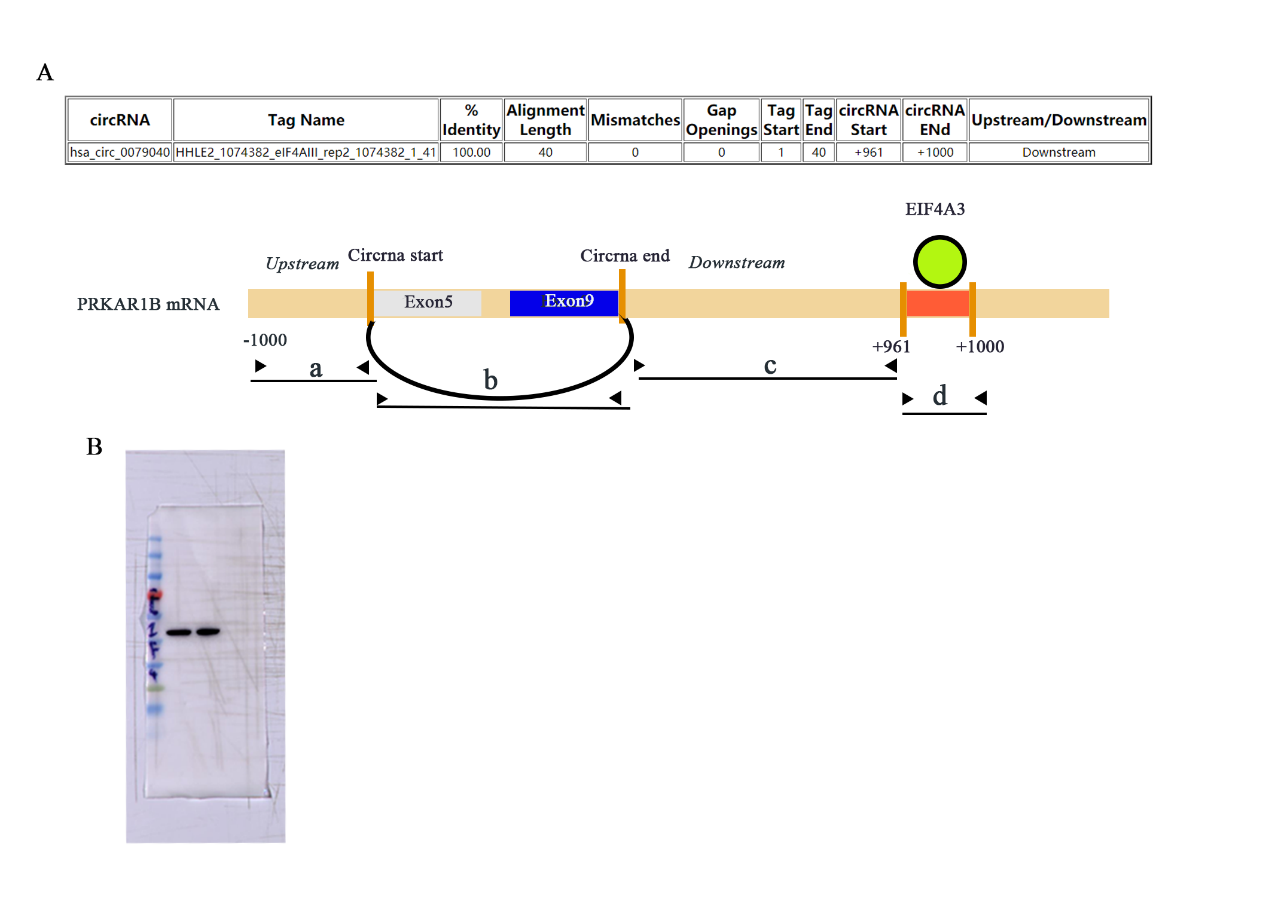
**

**Figure s1. EIF4A3 binds to the downstream of circPRKAR1B.**

**A** The binding sites of EIF4A3 in the upstream and downstream regions of the PRKAR1B mRNA transcript were predicted from the circRNA interactome. **B** the whole image of the blot.


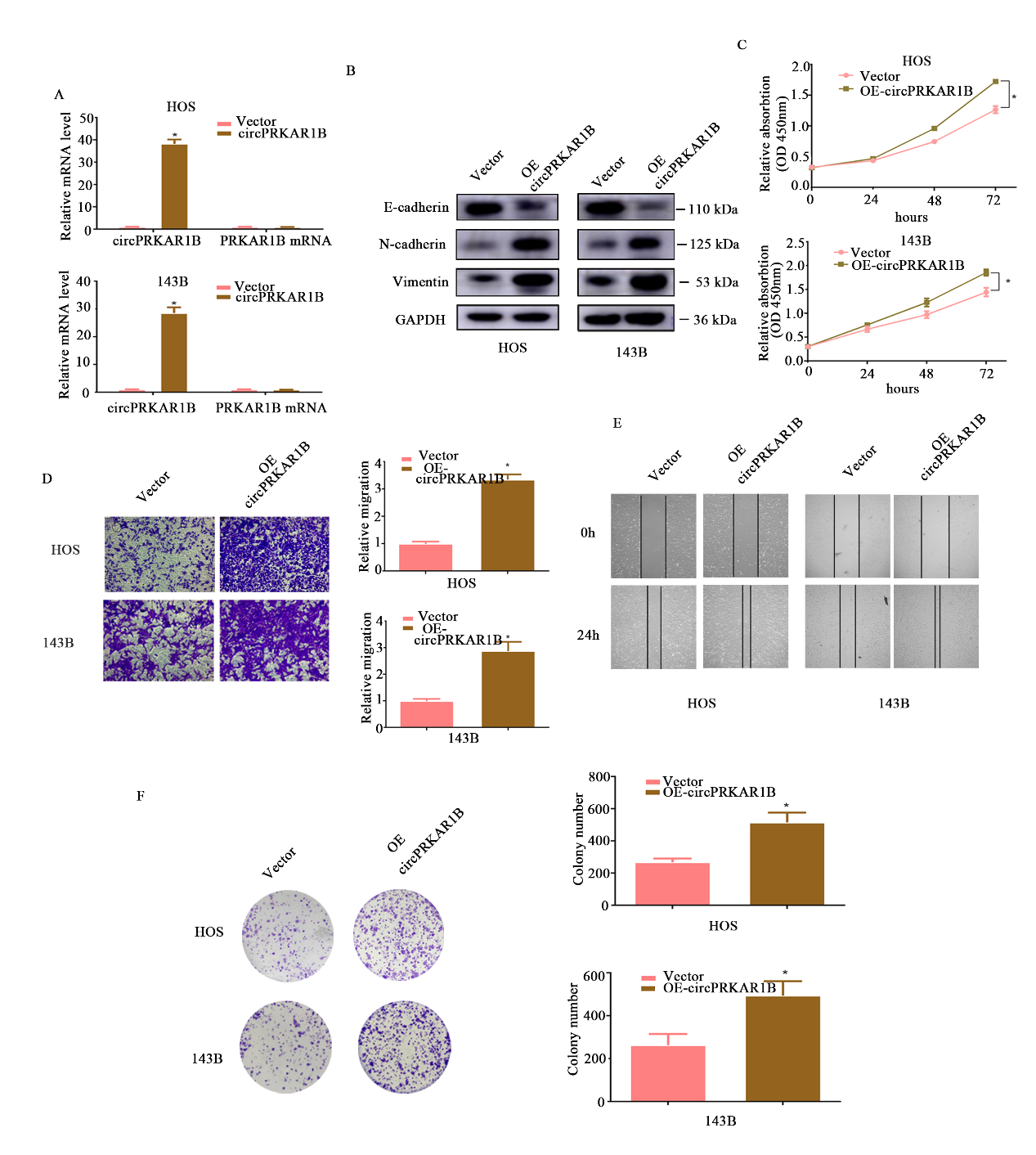


**Figure s2. circPRKAR1B overexpression promotes the migration, proliferation and EMT of OS cells.**

**A** HOS and 143B cells were stably transfected with a circPRKAR1B overexpression (OE) plasmid or vector plasmid, and the expression levels of circPRKAR1B and PRKAR1B mRNA were measured by RT-qPCR. **B** The protein expression of N-cadherin, E-cadherin, and Vimentin was measured by western blot analysis in both HOS and 143B cells transfected with the circPRKAR1B-OE plasmid. **C** Proliferation of HOS and 143B cells transfected with the circPRKAR1B-OE plasmid was measured by the CCK-8 assay. **D** The cell migration abilities of HOS and 143B cells transfected with circPRKAR1B-OE plasmid. **E** Representative images showing the effect of circPRKAR1B-OE on migration ability, as demonstrated by the wound-healing assay. **F** Colony formation assay showing changes in the proliferative capacity of OS cells stably transfected with Vector or circPRKAR1B-OE plasmid.

**
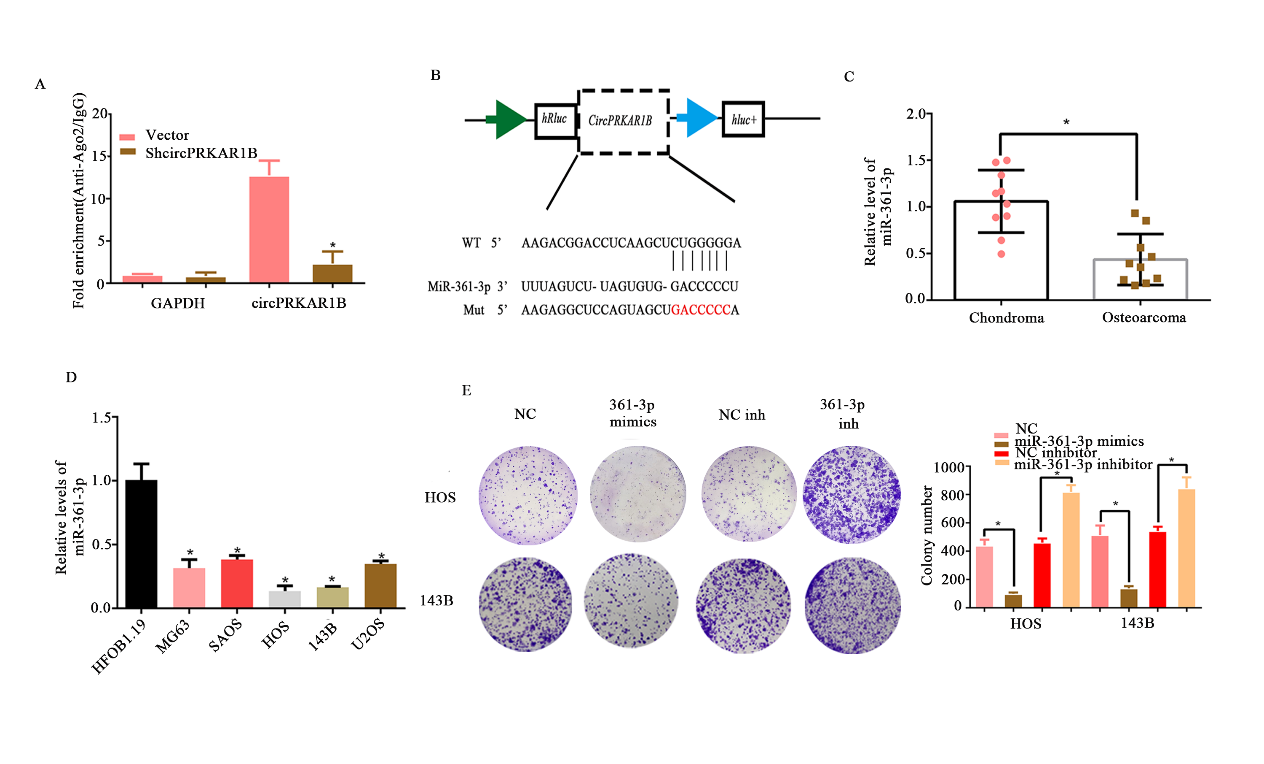
**

**Figure s3. The role of circPRKAR1B as a sponge for miR-361-3p, which inhibits the degree of OS malignancy**

**A** RIP analysis of circPRKAR1B levels in HEK-293 cells transfected with Ago2. **B** Schematic of dual-luciferase reporters (hFLuc-XbaL-hRLuc) carrying the wild-type or mutant circPRKAR1B sequence. **C** Low levels of miR-361-3p were expressed in human OS tissues compared with chondroma tissues (n = 30). **D** Expression of miR-361-3p in OS cells compared with control cells (HFOB1.19 cells). **E** Representative images of the colony formation assay showing changes in the proliferation ability of stably transfected OS cells. *p < 0.05. The data are presented as the means ± SD.


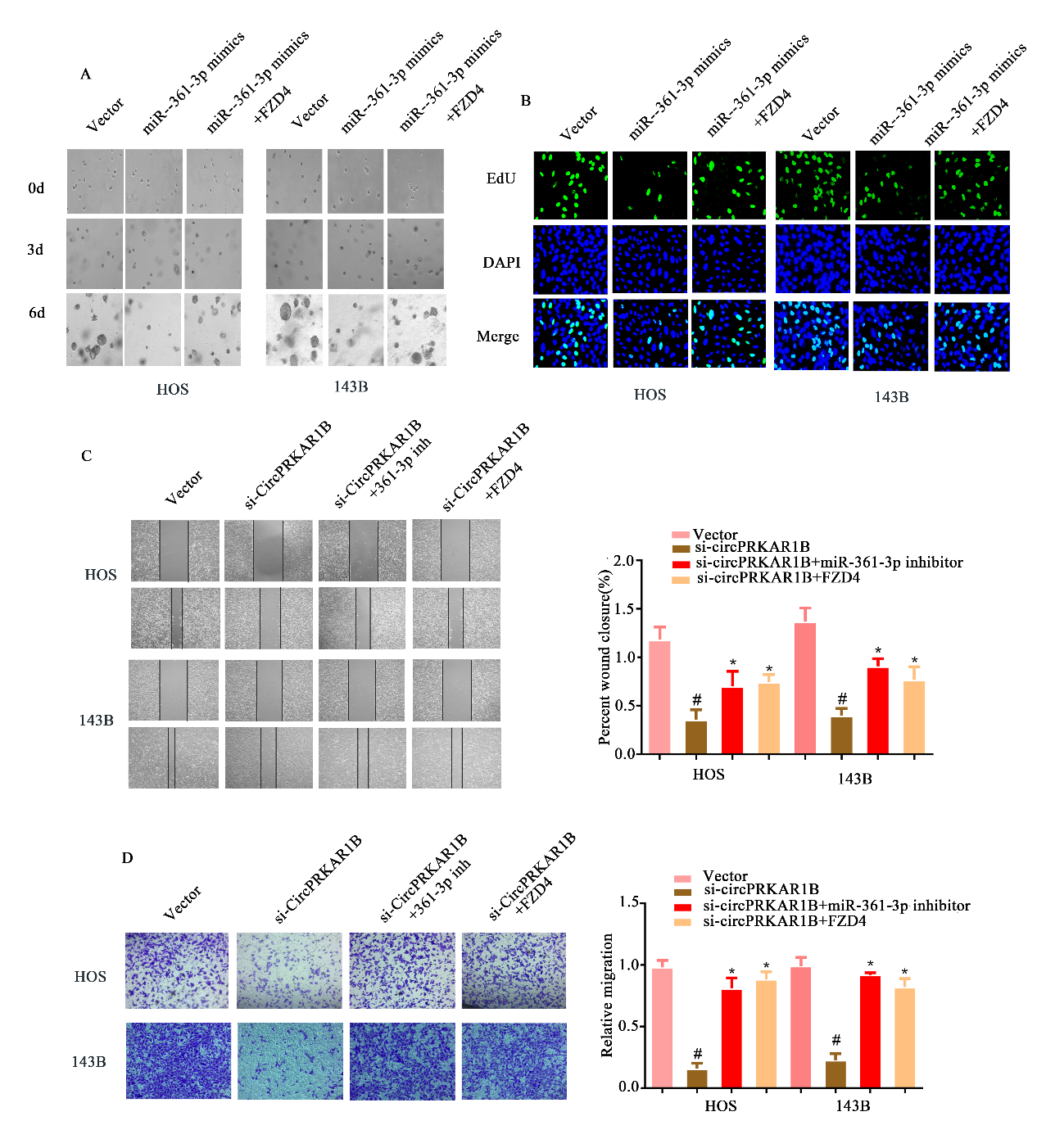


**Figure s4. The role of the circPRKAR1B/miR-361-3p/FZD4/Wnt/β-catenin axis in OS progression.**

A The reversal effect of FZD4 overexpression on the anchorage-independent colony-forming ability of OS cells transfected with miR-361-3p mimics. **B** The rescue effect of FZD4 on miR-361-3p mimic-transfected OS cell proliferation, as determined by the EdU assay. Nuclei were stained with DAPI, and the combination of EdU and DAPI indicated cells in S phase. **C** The effect of miR-361-3p and the rescue effect of FZD4 on migration were evaluated by the wound-healing assay in HOS and 143B cells. **D** Migration abilities of HOS and 143B cells transfected with miR-361-3p mimics or co-transfected with miR-361-3p mimics and the FZD4-OE plasmid were evaluated by Transwell migration assays. The data represent the mean ± SD (n = 3) (* P < 0.05).
